# Recent Advances in *Medicago truncatula* Genomics

**DOI:** 10.1155/2008/256597

**Published:** 2007-12-12

**Authors:** Jean-Michel Ané, Hongyan Zhu, Julia Frugoli

**Affiliations:** ^1^ Department of Agronomy, University of Wisconsin, Madison, WI 53706, USA; ^2^Department of Plant and Soil Sciences, University of Kentucky, Lexington, KY 40546, USA; ^3^Department of Genetics and Biochemistry, Clemson University, 100 Jordan Hall, Clemson, SC 29634, USA

## Abstract

Legume rotation has allowed a consistent increase in crop yield and consequently in human population since the antiquity. Legumes will also be instrumental in our ability to maintain the sustainability of our agriculture while facing the challenges of increasing food and biofuel demand. *Medicago truncatula* and *Lotus japonicus* have emerged during the last decade as two major model systems for legume biology. Initially developed to dissect plant-microbe symbiotic interactions and especially legume nodulation, these two models are now widely used in a variety of biological fields from plant physiology and development to population genetics and structural genomics. This review highlights the genetic and genomic tools available to the *M. truncatula* community. Comparative genomic approaches to transfer biological information between model systems and legume crops are also discussed.

## 1. INTRODUCTION

Legumes are usually defined by their typical flower structure and the ability of many of them to form root nodules in presence of symbiotic bacteria named rhizobia. With more than 18 000 species, legumes are found from the artic circle to the tropics and include many crops of agronomic importance for grain production, pasture, and forestry [1, 2]. The ability of more than 88% of legumes to obtain nitrogen from the air through root nodules was probably a major determinant in this evolutionary, ecological, and economical success [3]. Interestingly, the study of symbiotic associations with rhizobia as well as with arbuscular mycorrhizal (AM) fungi also drove the development of two model legumes: *Medicago truncatula* Gaertner and *Lotus japonicus* (Regel) K. Larsen.

While *M. truncatula* is an annual medic from the Trifolieae tribe and a close relative of alfalfa and clovers, *L. japonicus* belongs to the Loteae and is more distant from cultivated cool season legumes than *M. truncatula*. This phylogenetic distance to economically important crops is critical in the choice of *M. truncatula* by many researchers and support by numerous funding agencies. The use of both model legumes allows unique comparative genomic studies within the legume family as well as the comparison between two patterns of root nodule development: indeterminate with a persistent nodule meristem in the case of *M. truncatula* and determinate in *L. japonicus*. Unfortunately, these two models belong to the same cool season legumes (Galegoid clade), whereas soybean and common bean are tropical season legumes (Phaseolid clade). Soybean is therefore proposed as a third model legume for both its own economic weight and the phylogenetic proximity to other important crops [4, 5].

Research efforts on model legumes and especially on *M. truncatula* encompass a broad range of fields in plant biology from population biology [6–8] and plant development [9–16] to plant pathology [17–22], insect resistance [23–27], and biotechnology production [28]. The goal of this review is to provide an overview of the natural characteristics and genetic and genomic tools that make *M. truncatula* such a desirable experimental system for a growing number of plant biologists. We will highlight how information gained from *M. truncatula* can be transferred to other legumes crops through comparative genomics and we will share our vision of how *M. truncatula* can allow us to reach the goal of sustainable well-being through sustainable food and biofuel production.

## 2. *MEDICAGO TRUNCATULA* AS A MODEL LEGUME

Natural attributes of *M. truncatula* that make it a valuable genetic model include its annual habit and rapid life cycle, its diploid (2*n* = 16) and autogamous nature, its prolific seed production, and a relatively small genome of about 550 Mb. Jemalong A17 has been selected by the research community as a reference line for most genetic and genomic approaches and is derived from the major commercial cultivar. *M. truncatula* is native to the Mediterranean basin and is found in a wide range of habitats. It is therefore not surprising to find a high level of variation among and within natural populations [29]. Using microsatellite markers, a publicly available core-collection of 346 inbred lines was developed and thus represents the breadth of this natural diversity [30]. *M. truncatula* is used as a fodder crop in ley-farming systems in Australia, and a large and diverse collection is housed at the South Australian Research and Development Institute (SARDI) [8].

Like many higher plants, *M. truncatula* forms symbiotic associations with a wide array of arbuscular mycorrhizal (AM) fungi. As a legume, *M. truncatula* is also able to develop root nodules with *Sinorhizobium meliloti,* which is one of the best-characterized rhizobium species at the genetic level [31]. Cultivation-independent techniques have been used to sample the diversity of microbes associated with *M. truncatula* roots at various developmental stages and they reveal an extremely dynamic genetic structure of its rhizosphere [32].

Mutagenesis approaches using ethyl methane sulfonate (EMS), gamma rays, and fast neutron bombardment (FNB) have generated large mutant populations of *M. truncatula* from which mutants affected in symbiotic as well as developmental pathways have been identified [33–37]. T-DNA and *Tnt1* mutagenesis have been developed recently to generate tagged mutants for forward and reverse genetics purposes [38–40].

Several protocols have been optimized to transform *M. truncatula* using *Agrobacterium tumefaciens* [41–45]. These protocols are particularly efficient for the R108 and Jemalong 2HA lines but the regeneration efficiency still needs to be improved for Jemalong A17. This moderate efficiency as well as the time required for the regeneration steps is driving the preference of the *Medicago* community towards *Tnt1* versus T-DNA for gene tagging approaches [46] as well as the search for alternative transformation systems.

Hairy root transformation via *Agrobacterium rhizogenes* proved to be a rapid and efficient transformation system allowing the generation of transgenic roots in 2-3 weeks. Such “hairy roots” can be infected by rhizobia or AM fungi with symbiotic phenotypes indistinguishable from nontransgenic roots and are therefore an ideal system for plant-microbe symbiosis studies [47]. The development of DsRed as a visual reporter reduced the need for Kanamycin or Basta selection systems which were significantly decreasing nodulation efficiency. This hairy root transformation system is now used routinely to express protein fusions or RNA interference (RNAi) constructs [48–50]. The possibility to regenerate transgenic plants from hairy roots of the R108 line has been reported recently. This flexible approach should allow a rapid initial screening of phenotypes on hairy roots and a subsequent regeneration of transgenic plants if necessary [41]. An interesting ex vitro procedure that eliminates the need for labor-intensive in vitro culture will undoubtedly increase the throughput of hairy root transformations to a level compatible with genomic studies [51].

The *Medicago* community has therefore identified many ecotypes and developed a wide range of mutants and transgenic lines. A current goal of the International *Medicago truncatula* steering committee is to address the need for a stock center able to maintain, amplify, and distribute these lines to an ever growing community.

## 3. MAPPING THE GENOME OF *MEDICAGO TRUNCATULA*

**Figure 1:**
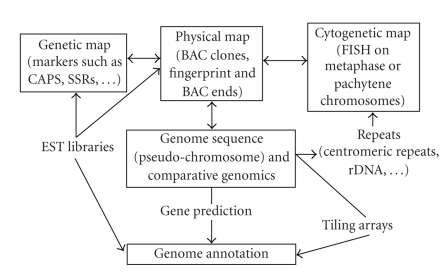
Integration of different maps and libraries to generate and annotate the genomic sequence of *M. truncatula.* Expressed sequence tags (EST) are used to generate genetic markers and to identify BAC clones in gene-rich regions as well as for gene identification. Repeats identified via genome sequencing and comparison with other species can be mapped via FISH on chromosome spreads.

Genetic and cytogenetic tools have been instrumental to the development of a “gene rich” genome sequence for *M. truncatula*. This project also required several bacterial artificial chromosomes (BAC) libraries that were developed using *Hin*dIII and *Eco*RI partial digests as well as a robust physical map (Figure 1).

Genetic maps have been developed from F2 populations and a wide array of genetic markers such as CAPS, AFLPs, RAPDs, and microsatellites (SSRs) [52–54]. One of them, based on a Jemalong A17 A17 × A20 F2 population, is currently used as a reference for the genome sequencing project (http://www.medicago.org/genome/map.php). Unfortunately, these F2 populations are either based on a limited amount of genomic DNA or require a labor-intensive vegetative propagation of F2 individuals. In order to provide sustainable tools to the community, genetic maps based on recombinant inbred lines (RILs) and highly polymorphic microsatellite markers are developed and will undoubtedly represent the future reference for *M. truncatula* genetics (T. Huguet, personal communication).

Cytogenetic maps based on fluorescence *in situ* hybridization (FISH) with interphase or metaphase chromosomes provide a quick access to the chromosomal location of BAC clones and repeated sequences [55–57]. Obtaining pachytene chromosomes is more labor intensive than metaphase chromosomes but provides an unequalled resolution all along the chromosome and particularly in euchromatic regions [58]. Information from such cytogenetic tools was instrumental for comparative genomics and map-based cloning projects but also allowed the determination that *M. truncatula* heterochromatin was mostly localized in pericentromeric regions. Genetic and cytogenetic markers corresponding to the borders of these regions have been developed [57, 59, 60]. Based on this unique chromosomal structure, it is therefore possible to predict through the genetic map if a BAC clone belongs to a euchromatic or a heterochromatic region. This observation as well as the possibility to select EST-rich BAC clones led the *M. truncatula* community to initiate the sequencing of euchromatic (gene-rich) regions via a BAC-by-BAC strategy (http://www.medicago.org/genome/).

Four centers share the sequencing effort of the 8 chromosomes: Bruce Roe et al. at the University of Oklahoma, Chris Town et al. at The Institute for Genomic Research (TIGR), Jane Rogers et al. at the Sanger Centre, and Francis Quétier et al. at the Genoscope. A physical map grouping and ordering of BAC clones was developed by the laboratory of Douglas R. Cook by combining *Hin*dIII digestion fingerprints with BAC-end sequence data through the FPC software [52, 61, 62]. More than 1370 FPC contigs cover 466 Mbp (93% of the genome) and are used to determine the minimum tiling path of gene-rich regions for whole genome sequencing [52].

As of February 2007, 188 Mb of genome sequence from 1950 BAC clones are publicly available. About 10% of this information is redundant due to the overlap of BAC clones necessary to create a tiling path and more than 300 gaps between contigs need to be filled. These gaps are sized by FISH and covered with contigs by long-range PCR or classical chromosome walking [62].

Integration of genetic, cytogenetic, physical, and sequence maps allowed the development of pseudochromosomes and greatly facilitated comparative mapping [52, 58–60]. Annotating pseudochromosome sequences is classically achieved through gene prediction programs and comparison with EST databases (Figure 1). The IMGAG (International *Medicago* Genome Annotation Group) has developed a unique automated pipeline to predict gene structures and functions [63]. More than 25 000 genes have been predicted so far and techniques to test these predictions need to be developed.

Oligonucleotides covering the entire sequence of pseudochromosomes can be printed on glass slides to generate tiling arrays. These arrays can be used for a wide range of applications from gene identification and detection of alternative splicing to comparative genome hybridization (CGH) and chromatin immunoprecipitation on chips (ChIP chips) [64–67]. 

## 4. SYSTEMS ANALYSIS

### 4.1. Transcriptomics

Large-scale EST sequencing is essential for functional genomics studies, permitting the direct identification of large gene collections and setting the stage for further analysis, such as those using DNA microarray technology. Several large EST projects have been completed [68–71]. The analysis of the almost 200 000 ESTs isolated from many different libraries constructed from diverse stages and treatments that came out of these projects is facilitated by searchable databases such as MtDB2 [72] and the TIGR Gene Index (http://www.tigr.org).

Both microarray and macroarray analyses of gene expression changes during symbiosis have been published [73–78]. These experiments ranged from analysis of a few thousand genes on filters during AM symbiosis [73] to almost 10 000 genes compared between wild type and nonnodulating mutants [76, 77] or between fix-mutants [79]. A dual symbiosis chip containing 10 000 *M. truncatula* genes and the entire *S. meliloti* prokaryotic genome allows side by side analysis of both partners in the symbiosis [80], and an Affymetrix chip with bioinformatically optimized oligonucleotides representing 48 000 genes is available (http://www.affymetrix.com/support/technical/datasheets/medicago_datasheet.pdf). As genome sequencing continues, following the expression of all *M. truncatula* genes under varying conditions should soon be possible. Affymetrix placed probe sets for 1850 *M. sativa* transcripts on these chips to facilitate the study of closely related species such as *M. sativa*. The use of *M. truncatula* arrays for analysis of *M. sativa* (crop alfalfa) gene expression has proven effective [81, 82].

Other effective genomic approaches to transcriptional analysis utilized to date in *M. truncatula* include suppressive subtractive hybridization (SSH) and serial analysis of gene expression (SAGE). In SSH, suppressive PCR is used to both normalize the abundance of transcripts in individual libraries and enrich for transcripts unique to the library by subtracting sequences common to several libraries, with rare sequences being enriched up to 1000 folds [83]. This method has been used to identify AM specific transcripts [84] and transcripts specifically involved in the *S. meliloti* symbiosis [85]. SAGE is a method for comprehensive analysis of gene expression patterns using short sequence tags obtained from a unique position within each transcript (10–14 bp) to uniquely identify a transcript. The expression level of the corresponding transcript is determined by quantifying of the number of times a particular tag is observed [86]. Although no publications have arisen yet, a project applying SAGE to *M. truncatula* is underway at the Center for *Medicago* Genomics Research at the Nobel Foundation (http://www.noble.org/medicago/GEP.html).

### 4.2. Proteomics

Another complementary approach to identify import gene products involved in interesting processes is to look at changes in the protein complement of a genome that vary by cell or treatment. In order for proteomic approaches to be useful in a system, a large sequence resource is necessary to match the sequences of peptides generated in tryptic digests to their proteins of origin. The growing sequence resource in *M. truncatula* allows identification of proteins by their mass spectra, making proteomics an effective approach for *M. truncatula* and proteomics approaches have become quite popular. A comprehensive review of considerations important in proteomics technology and applications in *M. truncatula* and *Arabidopsis* was recently published [87, 88]. Because small peptides have been shown to have roles in plant signaling, proteomics has been applied to identifying small protein/peptide components of certain *M. truncatula* tissues [89]. Proteomic approaches have also been applied to analyses of seed development [14, 16], pathogen interactions [90], symbiosome membranes [91], AM membranes [92], root microsomes [93], and other organ, tissue, and treatment-specific approaches [11, 94–100].

Most of the genes cloned thus far in the initial signal transduction pathway for nodulation are kinases [101]; suggesting global analysis of phosphoproteins is a way to identify important genes involved in signal transduction in *M. truncatula.* Unfortunately, phosphoproteins involved in cellular signaling are generally present in low abundance, creating new challenges for proteomics. By making adjustments the basic proteomics procedures, such as adding an enrichment step, a proof of concept experiment in *M. truncatula* phosphoproteomics, gives a taste of the potential of this approach [102].

### 4.3. Metabolomics

Alfalfa produces a number of secondary metabolites of great interest because of their contributions to human health and animal forage quality. The principle behind metabolomics is that metabolic profiling on a genomic scale offers a view of the metabolic status of an organism, which can lend insight to the study gene function or whole plant biology [103]. Successful attempts to link proteomics, transcriptomics, and metabolomics for cell cultures in *M. truncatula* have emerged from these studies [104, 105].

Metabolomics is a new and evolving science, and requires specialized equipment and multifaceted technical strategies. The Nobel Foundation employs a strategy that utilizes sequential or selective extraction followed by parallel analyses. The parallel analyses achieve a comprehensive view of the metabolome with high-performance liquid chromatography (HPLC), capillary electrophoresis (CE), gas chromatography (GC), mass spectrometry (MS), and various combinations of the above techniques such as GC/MS, LC/MS, and CE/MS. In addition to studying biological responses to biotic and abiotic elicitors in *M. truncatula* cellcultures, these techniques are being applied to the study of natural variants in *M. truncatula*, *M. truncatula* development, lignin biosynthesis, and legume-insect interactions.

Perhaps the most daunting aspect of metabolomic experiments is the analysis of the data. Early on, it became obvious that metabolomics required a standard similar to MIAME (minimum information about a microarray experiment) to allow comparison of data. A framework for the description of plant metabolomic experiments and their results has recently been developed. ArMet (architecture for metabolomics) is published and in accepted use [106, 107].

### 4.4. Phenomics

As more and more researchers use *M. truncatula* as a model, the need for a standardized method of describing phenotypes becomes acute. Since the timing and structure of vegetative and floral development in *M. truncatula* differ from *Arabidopsis*, adoption of standards such as those used for *Arabidopsis* [108] is inappropriate. Additionally, *M. truncatula* symbioses with AM fungi and *Sinorhizobium meliloti* add another dimension to developmental processes that require a standardized description of process stages and plant anatomy.

To date, a few attempts have been made to develop a standardized language for comparison. Vegetative growth parameters were carefully measured to provide a benchmark in [109], but the use of a glasshouse environment rather than a controlled light and temperature regime rendered the data not universally applicable. Likewise, flower development and response to vernalization have been documented in the same way [109], again in a glasshouse so the light intensity was uncontrolled. These experiments are progress toward a controlled standard for comparison of mutant phenotypes such as “late flowering” or “increased internodal distance.” Precision in phenotypic descriptions will be critical to genome scale mutant hunts.

There is no plant structural GO ontology terms for nodulation or nodule structures in the Plant Ontology Consortium site as of the February 2007 release (http://www.plantontology.org). The present plant ontology system provides terms for growth and developmental stages, as well as organs and tissues of *Arabidopsis*, maize and rice, but none of these plants nodulates, creating a problem for using GO annotation in *M. truncatula*.

### 4.5. Bioinformatics

All of the “omics” scale tools discussed above necessitate strong bioinformatics infrastructure for the species. A good place to begin is the *Medicago* Consortium website: http://www.medicago.org. In addition to a handbook of protocols for everything from growing and transforming *M. truncatula* to naming genes, links from this page lead to informatics tools such as ENSEMBL which allow a real time view of the annotation of the genome, tools allowing browsing of the genome for markers, genes, the location of BACs, the status of the sequencing project or the sequence status of any individual BAC. Users can also view the contigs assembled for sequencing, and make comparisons to other legumes through the legume information system [110] and the consensus legume database (www.legumes.org). Tools are also available through links from the medicago.org website for examining ESTs (TIGR, MtDB2, MENS), and in the future, examining microarray data. *In silico* approaches in *M. truncatula* have led to important insights, such as the identification of a large family of small legume-specific transcripts with conserved cysteine motifs whose function continues to be investigated [111–113].

But as genome scale biology is applied, the need to synthesize transcriptomics data, proteomics data, metabolomics data, and more becomes as important as the availability of informatics tools to analyze these data individually. Several steps in this direction have occurred within the *M. truncatula* community. Some of these integrated solutions are focused around a process, such as gene expression in mycorrhizal symbiosis [114]. Because of the nearly complete genome sequence and the cooperative nature of the sequencing and annotation of the genome, comprehensive integration of various data sources has been necessary from the beginning. Cannon et al. [115] provide a nice summary of the available sequence-based resources and how they interact. A freely available database of biochemical pathway data for *M. truncatula* (MedicCyc) contains more than 250 pathways with related genes, enzymes, and metabolites [116]. This provides the ability to not only visualize metabolomics data and integrate them with functional genomics data, but also allow comparison of *M. truncatula* pathways to those in other plants using the compatible AraCyc and RiceCyc databases.

## 5. REVERSE GENETICS

Reverse genetics approaches which identify mutants in a gene of interest based on sequence differences are critical genomic tools in a model system. A range of approaches are available, including retrotransposon tagging, T-DNA tagging, TILLING for EMS mutations, PCR screening for fast neutron mutations, and RNA-induced gene silencing (RNAi) [46]. Each method has advantages and disadvantages, and the choice of which method(s) to use will depend on the purpose of the investigator. In *M. truncatula*, RNAi, TILLING, and PCR screening of *Tnt1* insertion mutagenesis populations or fast-neutron generated deletion populations are reverse genetic approaches presently possible.

As noted above, the efficacy of RNAi in *M. truncatula* has been documented [48] including use in whole plants and in transformed roots. The combination of RNAi constructs and hairy root transformation is useful for large scale screening projects to identify genes of interest for further analysis. A large-scale project to identify gene function by silencing in *M. truncatula* is underway (http://www.cbs.umn.edu/labs/ganttlab/rnai.html). Initial results from this project include identification of a calcium-dependent protein kinase involved in nodule development, a gene that had not been identified through classical mutational analysis [117].

TILLING (Targeting Induced Local Lesions in Genomes) has proven useful in *Arabidopsis* and other plants (reviewed in [118]). Briefly, the sequence of a gene of interest is analyzed with a computer program that determines the consequences of all possible EMS mutations (primarily G to A transitions) on the amino acid sequence of the deduced protein. Regions are chosen for PCR amplification based on the concept that those regions most likely to result in altered protein function are highly conserved domains in proteins, and PCR primers are designed to amplify these regions of DNA from each plant. The PCR products are analyzed for single-base pair changes in a high throughput sequencing gel system using an enzyme that detects and cleaves single-base mismatches in DNA. The use of high throughput methodology and a well-characterized and curated population of mutagenized plants allows a plant containing a lesion in the gene of interest to be identified in days. The benefits of TILLING are not only the rapid identification of lesions, but the nature of the lesions themselves. The point mutations generated by EMS treatment allow the use of TILLING to generate an allelic series that includes both missense and nonsense mutations.

In *M. truncatula*, the Cook lab. at UC Davis developed a population of ∼4000 curated EMS mutagenized plants for purpose of TILLING. This resource is currently unavailable as a community resource due to the absence of funding. To date genotypic screens for mutations in 15 genes of interest to the Cook lab or collaborators have been undertaken, and 143 mutants identified, with recovery rates of 9.89 alleles per kbp screened. Phenotypic characterization of one (of 23 unique) allele identified from one of the early genotypic screens for mutations in the *M. truncatula* arbuscule specific phosphate transporter *MtPT4* is described by Javot et al. [119]. Characterization of other mutants in this collection is currently ongoing or advanced to the stage where manuscripts are in preparation for submission (Douglas R. Cook and Varma Penmetsa, personal communication).

A reverse-genetics platform has been established in *Medicago truncatula* exploiting fast neutron (FN) mutagenesis and a highly sensitive PCR-based detection first documented in *Arabidopsis* [120]. The FN-based screening platform produces complete loss of function mutants by identifying large deletions in the targeted region. Central to this platform is the development of a detection strategy which allows a mutant amplicon, possessing an internal deletion, to be preferentially amplified in pools where genomic target sequence is present at a 20 000-fold excess. This detection sensitivity has been achieved through a combination of techniques for suppressing the amplification of the wild-type sequence and preferentially amplifying the mutant product. The population has been arrayed such that 12 000 M2 plants can be analyzed in 4 PCR reactions. These megapools can then be dissected using 25 PCR reactions on 3D pools, allowing identification of the individual seed lot containing the mutant. In comparison with the well-established TILLING method [121–123], which utilizes 8-fold PCR-based screening, FN alleles can be isolated at a fraction of the cost and avoid the problems associated with EMS mutagenesis of targeting small genes and the very high number of background mutations in isolated mutants. An initial characterization of the FN system analyzed 10 genes in a subpopulation of 60 000 M2 plants. Mutants were recovered for 4 target genes. A population of 180 000 M2 plants has now been established and should allow the recovery of mutants from a majority of targeted loci. Information for accessing this resource can be found at www.jicgenomelab.co.uk. (C. Rodgers and G. E. D. Oldroyd, personal communication).

Recently, researchers have identified a tobacco retrotransposon, *Tnt1*, that moves randomly in *M. truncatula* but only upon passage through tissue culture [39]. This retrotransposon can be used to generate a large population of plants with tagged mutation sites in tissue culture that become stable upon regeneration of whole plants, an important resource for both forward and reverse genetics. A population mutagenized by *Tnt1* can be used for reverse genetic screens by sequencing of tagged sites and forward genetic screens by observation of phenotypes. The isolation of the *M. truncatula pim* gene through this reverse genetics approach demonstrates the utility of the system for identifying mutants by sequence [40].

## 6. TRANSLATIONAL GENOMICS FROM MODEL TO CROP LEGUMES

The value of the model systems will be enhanced by the ability to connect model systems to crops at the structural and functional genome levels. For example, conserved genome structure (synteny) between model and crop species could allow the use of model species as a surrogate genome for map-based cloning of agronomically important genes in crops with complex genomes. Moreover, detailed knowledge of the molecular basis of conserved phenotypes in model species can be translated to great advantage for gene discovery in related species. Working with *M. truncatula* as a reference system, researchers have used comparative genomics tools to bridge model and crop legumes through comparative mapping of orthologous genes [54, 124, 125]. Alignment of linkage maps and sequenced orthologous regions reveals an extensive network of macro- and microsynteny between legume species [125–127]. In fact, the conserved genome organization between *M. truncatula* and crop legumes has allowed for cross-species prediction and isolation of several genes required for root symbiosis using *M. truncatula* as a surrogate [128, 129] and reviewed in [125]. Despite the emerging picture of substantial synteny between legumes, the level of conservation decreases as the evolutionary distance increases [124, 125]. Thus, comparisons within Galegoid or Phaseolid legumes tend to reveal chromosome-level synteny, while comparisons between the two clades tend to reveal large-segment synteny, which is also reflected in the differences in chromosome number between Galegoid and Phaseolid legumes [125, 127].The broad taxonomic distance separating the two clades warrants the development of one or two reference systems within each clade, *M. truncatula,* and *L. japonicus* for the cool-season legumes and soybean for the tropical-season legumes [130].

A significant effort has been undertaken in comparative genomic analysis of legume resistance gene homologs (RGHs). Most plant disease resistance genes identified to date belong to the nucleotide binding site (NBS) leucine rich repeat (LRR) family [131]. NBS-LRR genes can be further classified by the presence or absence of a toll/interleukin receptor (TIR) homology domain. In previous studies, researchers investigated the genomic architecture of RGHs in *M. truncatula* [21], and used phylogenetic methods to assess evolutionary trends in this large gene family in legumes and across the angiosperms [21, 132]. The results from these studies revealed several important insights into RGH gene evolution in plants. Despite the presence of the two major lineages of RGHs (i.e., TIR and non-TIR NBS-LRR genes) in all dicots, each of these lineages is populated by numerous family-specific or family-predominant clades [132]. For example, the major RGH clades that define legumes are absent from the Brassicaceae and Solanaceae, and vice versa. Thus, there are likely to be aspects of RGHs (including both structural and functional attributes) that are peculiar to individual plant families. When phylogenetic analyses were conducted within the legume family [21], it was found that all known major clades in legumes are represented by sequences from *M. truncatula*, providing evidence that the major RGH radiations predate the respective speciation events. There are also cases that cophyletic RGHs occupy syntenic positions between legumes. The availability of a nearly complete catalog of *M. truncatula* NBS-LRR genes is expected to greatly enable rapid and efficient characterization of RGHs in other closely related legumes. A legume genome project towards this effort has recently been funded by the NSF Plant Genome Research Program. The goal of this funded project was to develop genomic tools for five less-studied legume species (i.e., chickpea, pigeon pea, cowpea, peanut, and lupine), which are economically important in the developing countries of Africa and Asia (D. R. Cook, personal communication).

Forage legumes, such as alfalfa, red clover, and white clover, are an important component of animal and sustainable agriculture throughout the world. In addition to providing superior forage quality for animal production and improving soil fertility through nitrogen fixation, forage legumes also contribute to the improvement of soil structure and control of soil erosion. Alfalfa (*Medicago sativa*), for example, is grown on over 26 million acres and ranks third in acreage planted and dollar value in the US (USDA Crop Values Summary 2005). The true clovers (*Trifolium* spp.), which are often grown together with forage grasses, are also widely distributed. Despite serving as a major source of meat and milk products via animals, the economic importance of forages to food production and the agricultural economy of the US are not fully appreciated. Consequently, forage legumes suffer from poorly developed genetic and genomic infrastructure due to both limited federal funding and their intractable genetic system (e.g., polyploidy and self-incompatibility). The lack of such infrastructure limits the application of genomics-enabled technologies in the genetic improvement of forage legumes. Nevertheless, all these forage legumes are closely related to the model legume *M. truncatula*, a cool-season legume within the tribe Trifolieae. Therefore, forage legumes could be an immediate beneficiary of the study of *M. truncatula* genomics. As many of the pathogens of *M. truncatula* are also pathogens of closely related forage legumes, it should be possible to clone resistance genes that are active against pathogens of crop legume species in *M. truncatula*. In addition, due to the close relationship of resistance gene sequences between these species, it is likely that functional resistance genes can be moved across species boundaries by transgenic approaches.

Thus the genetic, genomic, and molecular tools available in *M. truncatula* allow not only investigation of basic processes important to legumes, but also transfer of that information to important crop species.
